# Ginsenoside F1 Protects the Brain against Amyloid Beta-Induced Toxicity by Regulating IDE and NEP

**DOI:** 10.3390/life12010058

**Published:** 2022-01-01

**Authors:** Yee-Jin Yun, Bong-Hwan Park, Jingang Hou, Jung-Pyo Oh, Jin-Hee Han, Sun-Chang Kim

**Affiliations:** 1 Department of Biological Sciences, Korea Advanced Institute of Science and Technology, Daejeon 34141, Korea; yyj07@kaist.ac.kr (Y.-J.Y.); nice2996@kaist.ac.kr (J.-P.O.); han.jinhee@kaist.ac.kr (J.-H.H.); 2 Intelligent Synthetic Biology Center, Daejeon 34141, Korea; bhpark93@kaist.ac.kr (B.-H.P.); houjg2022@guest.kaist.ac.kr (J.H.)

**Keywords:** amyloid-β, blood–brain barrier, ginsenoside F1, insulin-degrading enzyme, neprilysin

## Abstract

Ginsenoside F1, the metabolite of Rg1, is one of the most important constituents of *Panax ginseng*. Although the effects of ginsenosides on amyloid beta (Aβ) aggregation in the brain are known, the role of ginsenoside F1 remains unclear. Here, we investigated the protective effect of ginsenoside F1 against Aβ aggregation in vivo and in vitro. Treatment with 2.5 μM ginsenoside F1 reduced Aβ-induced cytotoxicity by decreasing Aβ aggregation in mouse neuroblastoma neuro-2a (N2a) and human neuroblastoma SH-SY5Y neuronal cell lines. Western blotting, real-time PCR, and siRNA analysis revealed an increased level of insulin-degrading enzyme (IDE) and neprilysin (NEP). Furthermore, liquid chromatography with tandem mass spectrometry (LC-MS/MS) analysis confirmed that ginsenoside F1 could pass the blood–brain barrier within 2 h after administration. Immunostaining results indicate that ginsenoside F1 reduces Aβ plaques in the hippocampus of APPswe/PSEN1dE9 (APP/PS1) double-transgenic Alzheimer’s disease (AD) mice. Consistently, increased levels of IDE and NEP protein and mRNA were observed after the 8-week administration of 10 mg/kg/d ginsenoside F1. These data indicate that ginsenoside F1 is a promising therapeutic candidate for AD.

## 1. Introduction

Alzheimer’s disease (AD) is a classic cause of dementia. The pathological features of AD include memory loss and cognitive decline [1]. Major lesions are found in the cerebral cortex and hippocampus, which have abnormally hyperphosphorylated neurofibrillary tangles in the neurons and amyloid beta (Aβ) plaque accumulating outside the cell. These pathological characteristics are common, among which the toxic protein Aβ is a major component, and its over-accumulation is reported to be a common phenomenon [2,3].

Aβ peptides, the main components of Aβ plaques found in AD patients, are natural products of metabolism and contain 36–43 amino acids. Aβ peptides are derived from the β-site proteolytic cleavage of amyloid precursor protein (APP) by a β-secretase, and γ-secretase, a protein complex with presenilin 1 at its catalytic core [4]. Aβ aggregates to form soluble oligomers, which may exist in several forms. Accumulation of Aβ is toxic and leads to neuronal damage, which further leads to synaptic dysfunction and neurodegeneration. Aβ levels in the brain can be mediated by the dynamic equilibrium between Aβ production from APP and removal by amyloid-degrading enzymes (ADEs) [5]. ADE has received much attention due to its role in removing harmful amyloid moieties. In AD patients, the survival of the synapse and neuronal cell is directly influenced by insulin resistance and indirectly by insulin-degrading enzyme (IDE), which is likely a key player in Aβ catabolism [6]. In addition, neprilysin (NEP) inhibits the progression of AD by degrading Aβ plaques [7].

Ginseng, used in traditional medicine in East Asian countries, has a wide range of pharmacological effects and has been used to treat many diseases, particularly those associated with aging and memory loss [8]. Ginsenosides are reported to have anti-cancer, anti-allergic, anti-inflammatory, and antioxidant effects [9,10,11,12]. Ginsenoside Rg1, the major bioactive component of *Panax ginseng*, is used for treating diseases of the central nervous system (CNS), such as cerebral edema and cerebral ischemia. Ginsenoside Rg1 cannot pass through the blood–brain barrier (BBB) [13,14]. Ginsenoside F1 is barely contained in Korean red ginseng and commonly metabolized via deglycosylation by intestinal microflora from the Re and Rg1 [15]. Ginsenoside F1 production can be used by fermentation or enzymatic methods to remove and convert the glucose moiety by converting the ginsenoside Rg1 or Re with a glucose residue at the C6 position. Although studies have demonstrated the biological functions of F1, such as an anti-cancer, immune modulatory, anti-aging, and anti-inflammatory effects, its role in brain health remain unknown.

In this study, we investigated whether ginsenoside F1 reduces Aβ_1–42_ levels in AD. We showed that ginsenoside F1 inhibits Aβ aggregation-induced cytotoxicity and reduces Aβ levels by upregulating the expression of IDE and NEP. We found that ginsenoside F1 passes through the BBB and inhibited the increase of Aβ plaques. To the best of our knowledge, this study is the first to provide evidence that ginsenoside F1 ameliorates AD symptoms through regulation IDE and NEP. These findings suggest that ginsenoside F1 protects cells from Aβ-induced toxicity in AD.

## 2. Materials and Methods

### 2.1. Materials

Dulbecco’s modified Eagle’s medium (DMEM), fetal bovine serum (FBS), and phosphate-buffered saline, DMEM/F12, OPTI-MEM, and penicillin-streptomycin solution were purchased from Thermo Fisher Company. trichloroacetic acid (TCA) and acetone were purchased from Fluka (Buchs, Switzerland); Aβ was obtained from Cellmano Biotech (Hefei, China); horseradish peroxidase (HRP)-linked anti-rabbit IgG and HRP-linked anti-mouse IgG were bought from Cell Signaling Technology (Beverly, MA, USA); IDE and NEP from Abcam (La Jolla, CA, USA); Aβ and β-actin were purchased from Santa Cruz Biotechnology (Santa Cruz, CA, USA). All chemicals were of the highest commercially available grade. Ginsenoside F1 (>95% pure) was prepared using an enzymatic method from *Panax ginseng* extract as previously reported [16].

### 2.2. Cell Culture

Mouse neuroblastoma neuro-2a (N2a) cells and Human neuroblastoma SH-SY5Y cells were purchased from ATCC (Manassas, VA, USA) and cultured as manual provided by manufacture.

### 2.3. Oligomerization of Aβ_1–42_

Lyophilized powder of Aβ_1–42_ was processed with HFIP and dissolved in DMSO to obtain a concentration of 5mM, then sonicated in the water bath for 10 min to ensure complete suspension. Diluted monomeric aliquot with sterile phosphate buffer to a final concentration of 100 μM, vortexed for 30 s, and incubated for 24 h at 4 °C to obtain oligomeric Aβ_1–42_.

### 2.4. Measurement of Cell Viability and Cytotoxicity

N2a (2 × 10^5^ cells/mL) and SH-SY5Y (2 × 10^5^ cells/mL) cells were cultured in 48-well plates for 24 h. Then, N2a and SH-SY5Y cells were treated with ginsenoside F1 (2.5–10 μM), aggregated Aβ (10 μM), or ginsenoside F1 (2.5–10 μM) and aggregated Aβ_1–42_ (10 μM) for 24 h at 37 °C and 5% CO_2_. After incubation, the cells were treated with the MTT solution (final concentration, 1 mg/mL) for 1 h. Absorbance at 550 nm of the dark blue formazan crystals formed was quantified using a spectrophotometer (I-mark, Bio-Rad, Hercules, CA, USA). LDH levels in the supernatants were measured using a colorimetric test based on NADH concentration. The assay was performed using a microplate reader (Spark 10 M, Tecan, Männedorf, Switzerland).

### 2.5. Trichloroacetic Acid Precipitation

Culture medium was diluted 4:1 with TCA. Incubate mixture for 10 min at 4 °C and centrifuge at 10,000× *g* for 5 min at 4 °C. Remove supernatant and wash the pellet with 200 μL of 90% cold acetone for three times. Remove supernatant and the pellet containing IDE and NEP was air-dried.

### 2.6. RNA Preparation and Quantitative Real-Time PCR

N2a and SH-SY5Y cells were treated with ginsenoside F1 (0, 1, 2.5, 5, and 10 μM) for 24 h at 37 °C and 5% CO_2_. Total RNA was extracted from ginsenoside F1-treated or untreated cells using an RNA isolation kit (Takara Shuzo, Kyoto, Japan), analyzed by qRT-PCR using Quantitect SYBR Green RT-PCR (Qiagen, Hilden, Germany) on a CFX96 system (Bio-Rad), and normalized to β-actin. The primers are listed in Supplementary Table S1.

### 2.7. Western Blot Analysis

Proteins were extracted and concentrations were determined using a BCA protein assay kit (Pierce, Rockford, IL, USA). Total of 40 µg protein were heated for 5 min and separated by 10% SDS-PAGE, and then transferred to PVDF membranes. Following blocking with 1% bovine serum albumin (BSA) (Sigma, St. Louis, MO, USA) in TBS buffer and incubated overnight at 4 °C with the following primary antibodies: IDE, NEP (1:1000), and β-actin (1:2000, as loading control). HRP-conjugated goat anti-rabbit IgG (Cell signaling) was used as a secondary antibody. Finally visualized using the enhanced chemiluminescence (ECL) solution (Pierce Biotechnology, Waltham, MA, USA). Signals were detected using ChemiDoc (Bio-rad) and analyzed with Image J software (NIH, Bethesda, ME, USA). Original data are represented in Table S2.

### 2.8. Transfection with Small Interfering RNA

IDE and NEP small interfering RNA (siRNA) were obtained from Santa Cruz Biotechnology, Inc. (Santa Cruz, CA, USA). Cells were grown to 50% confluence and transfected using the Lipofectamine 2000 (Invitrogen, Carlsbad, CA, USA) with IDE siRNA or NEP siRNA according to the manufacturer’s instructions.

### 2.9. ELISA

N2a and SH-SY5Y cells were treated with aggregated Aβ (10 μM) alone or without aggregated Aβ for 1 h and then replace with conditioned medium containing ginsenoside F1 (0, 1, 2.5, 5, and 10 μM) or ginsenoside Rg1 (10 μM) and incubated at 37 °C and 5% CO_2_ for 24 h. The medium was collected, treated with protease inhibitors from Calbiochem (La Jolla, CA, USA), and cleared by centrifugation (10,000× *g*, 5 min, 4 °C). Aβ_1–42_ contents were determined by using a commercially available kit (Thermo Fisher Scientific, Waltham, MA, USA) according to the manufacturer’s instructions.

### 2.10. Thioflavin-T Assay

N2a and SH-SY5Y cells treated with aggregated Aβ (10 μM) were incubated with or without ginsenoside F1 (2.5 μM) or ginsenoside Rg1 (2.5 μM) at 37 °C and 5% CO_2_ for 24 h. Samples (20 μL) were collected from the incubation mixture and added to 980 μL thioflavin-T solution. Averages of emission fluorescence were plotted as a function of time.

### 2.11. Animals

APPswe/PSEN1dE9 double-transgenic AD mice with a B6 × C3 background and B6 × C3 wild-type mice were purchased from The Jackson Laboratory (MMRRC stock no. 034829-JAX). All procedures and protocols were approved by the Animal Ethics Committee of the Korea Advanced Institute of Science and Technology. All experiments were performed in accordance with the guidelines of the Institutional Animal Care and Use Committee. To test the effect of ginsenoside F1 on AD, 10 mg/kg/d ginsenoside F1 was orally administered through a gelatin-based jelly, which was prepared as described previously [17]. After the 8-wk administration of ginsenoside F1, immunohistochemical analysis and western blotting were performed.

### 2.12. Liquid Chromatography with Tandem Mass Spectrometry (LC-MS/MS) Analysis

Plasma and tissue samples were analyzed using LC-MS/MS. The system was composed of a NEXERA SIL-30AC apparatus (Shimadzu Co., Kyoto, Japan) with a 100 mm × 2.1 mm, 2.6 μm, Kinetex C_18_ column (Phenomenex, Torrance, CA, USA) coupled to a Triple QUAD 3500 mass spectrometer (AB Sciex Co., Concord, ON, Canada), equipped with an electrospray ionization source. The parameters of the triple QUAD 3500 mass spectrometer were as follows: ionspray voltage −4200V, ion source gas 1, curtain gas 20, and collision gas 2. Values for the declustering potential, focusing potential, entrance potential, collision cell exit potential, and collision energy varied with respect to the measured ginsenosides. For full-scan MS analysis, the spectra were recorded in the *m*/*z* range of 400–1000.

### 2.13. Histology and Staining

Mouse brains were dissected and fixed overnight in 4% paraformaldehyde in PBS (pH 7.4) and incubated in 30% sucrose for 48 h. Brains were frozen in optimal cutting temperature compound and sectioned at a thickness of 30-μm. Wash the section with PBS, and incubated for 10 min in 3% H_2_O_2_ in PBS. Sections were blocked for 20 min and incubated with mouse Aβ (clone 6E10, Covance) diluted 1:750 overnight at 4 °C with agitation. Lastly, sections were washed and incubated with anti-mouse immPRESS (ready to use, Vector Laboratories) for 30 min at room temperature. Sections were mounted on slides and counterstained with Mayer’s hematoxylin.

### 2.14. Statistical Analysis

Statistical analyses were performed using Prism software (GraphPad 8). Direct comparisons within single genotype groups were performed by using one-way ANOVA followed by Tukey’s post-hoc test or Student’s *t*-test. Statistical differences were considered significant at * *p* < 0.1. ** *p* < 0.01, *** *p* < 0.001, **** *p* < 0.001. All experiments were performed independently at least three times; the results are expressed as mean ± s.e.m

## 3. Results

### 3.1. Ginsenoside F1 Reduces Aβ_1–42_-Induced Cytotoxicity in Neuronal Cells

**Figure 1:**
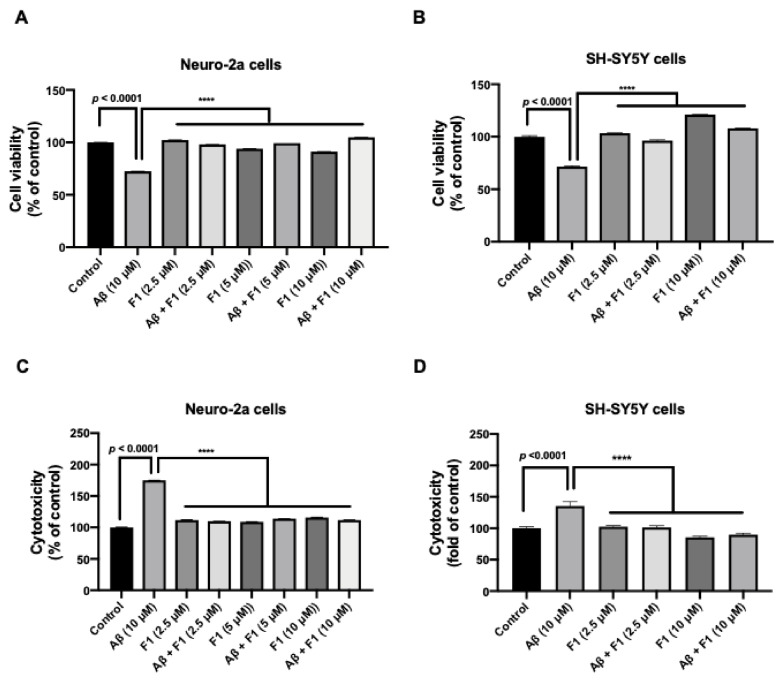
Ginsenoside F1 protects cells from aggregated amyloid β (Aβ)-induced toxicity in vitro. Mouse neuroblastoma neuro-2a (N2a) and human neuroblastoma SH-SY5Y cells were incubated with ginsenoside F1 (2.5–10 μM), aggregated Aβ_1–42_ (10 μM), or ginsenoside F1 (2.5–10 μM) and aggregated Aβ_1–42_ (10 μM) for 24 h at 37 °C, 5% CO_2_. (**A**,**B**) Cell viability was quantified using the MTT assay. (**C**,**D**) Cytotoxicity was measured using the LDH assay. All experiments were performed in triplicate, and data are mean ± SEM **** *p* < 0.0001.

The hallmarks of AD include the formation of Aβ plaques and neurofibrillary tangles and extensive neuronal degeneration [18]. The molecular target of Aβ toxicity is unknown. We investigated whether ginsenoside F1 affects cell viability in neuronal cells. Cell viability was examined using MTT assay. Neuronal cells treated with ginsenoside F1 without Aβ had a similar proliferation value as control cells, which was significantly higher than that for Aβ_1–42_-treated N2a and SH-SY5Y cells (Figure 1A,B). In addition, Aβ_1–42_-induced toxicity was investigated using LDH assay. Ginsenoside F1 (2.5–10 μM) protected neuronal cells from aggregated Aβ_1–42_-induced toxicity (Figure 1C,D). Thus, ginsenoside F1 significantly reduced Aβ_1–42_-induced cytotoxicity in neuronal cells.

### 3.2. Ginsenoside F1 Treatment Reduces the Secretion of Aβ_1–42_ in Neuronal Cells

**Figure 2:**
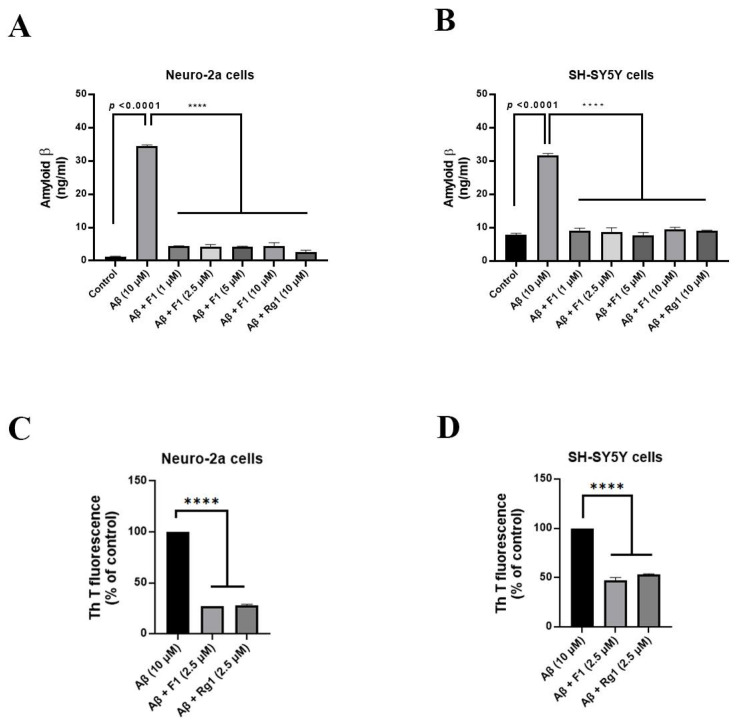
Ginsenoside F1 reduces amyloid β (Aβ) production and secretion in vitro. Neuro-2a and SH-SY5Y cells treated with aggregated Aβ_1–42_ (10 μM) were incubated with or without various concentrations of ginsenoside F1 (0, 1, 2.5, 5, and 10 μM) for 24 h at 37 °C, 5% CO_2_ and analyzed by ELISA and Thioflavin T (ThT) assays. (**A**,**B**) Quantification of ELISA assay. (**C**,**D**) Quantification of ThT assay. All experiments were performed in triplicate, and data are mean ± s.e.m. **** *p* < 0.0001.

AD involves Aβ-induced neuronal death, and extracellular Aβ toxicity contributes to neuronal loss in AD. We evaluated the secretion of Aβ_1–42_ plaques using enzyme-linked immunosorbent assay (ELISA). Treatment with ginsenoside F1 (1–10 μM) reduced Aβ_1–42_ secretion in N2a (Figure 2A) and SH-SY5Y (Figure 2B) cells in a concentration-dependent manner. Moreover, we performed the thioflavin-T fluorescence assay to investigate whether ginsenoside F1 reduces Aβ_1–42_ plaque formation in neuronal cells. Treatment of neuronal cells with ginsenoside F1 (2.5 μM) for 24 h decreased Aβ_1–42_ plaque formation (Figure 2C,D).

### 3.3. Ginsenoside F1 Upregulates the Expression of Aβ-Degrading Peptidases in Neuronal Cells

**Figure 3:**
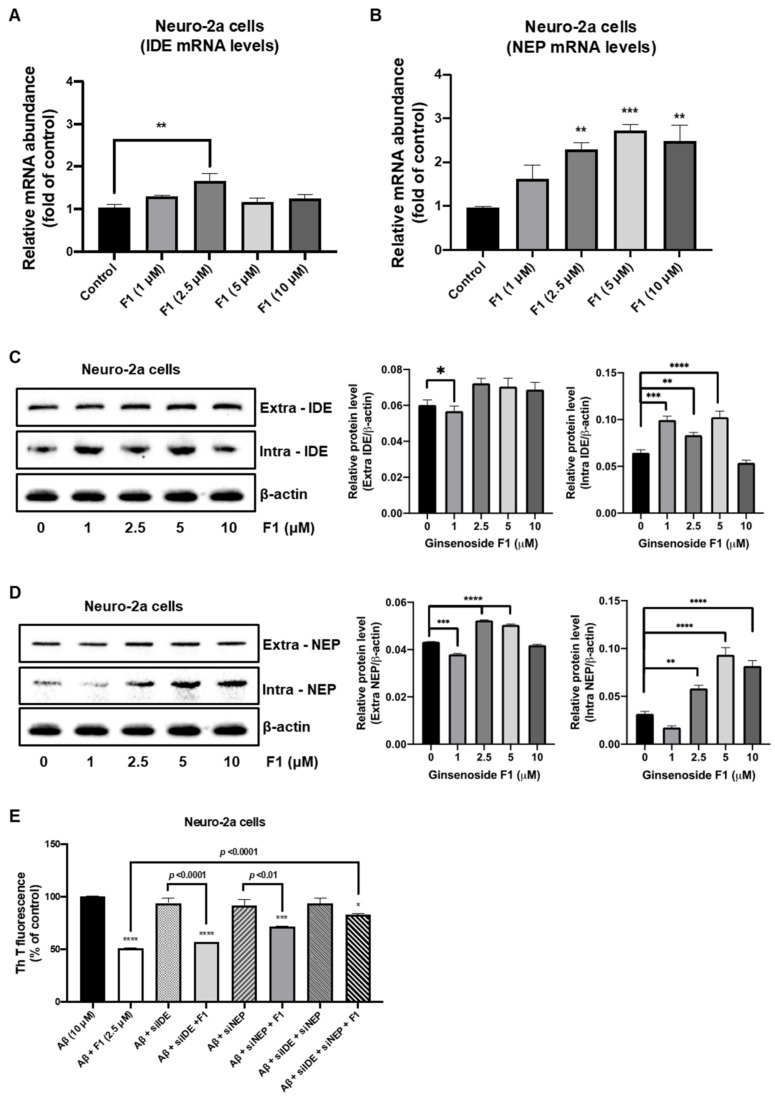
Ginsenoside F1 is associated with amyloid β (Aβ) peptidases in N2a cells. (**A**,**B**) mRNA transcriptional levels of IDE and NEP were measured using real-time PCR analysis. (**C**,**D**) Protein expression levels of IDE and NEP were measured using Western blotting. (**E**) Aβ_1–42_ aggregation in neuro-2a cells was measured by thioflavin-T analysis. All experiments were performed in triplicate, and data are mean ± s.e.m. * *p* < 0.1, ** *p* < 0.01, *** *p* < 0.001, **** *p* < 0.0001.

**Figure 4:**
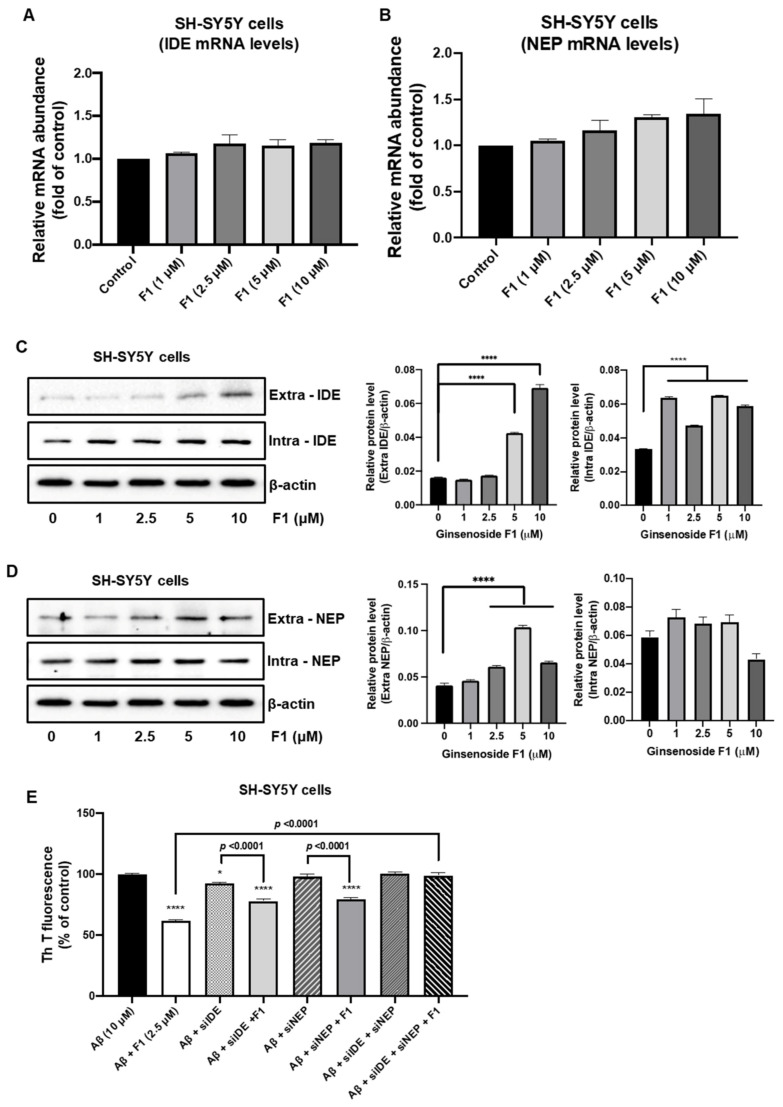
Ginsenoside F1 is associated with amyloid β (Aβ) peptidases in SH-SY5Y cells. (**A**,**B**) mRNA transcriptional levels of IDE and NEP were measured using real-time PCR analysis. (**C**,**D**) Protein expression levels of IDE and NEP. (**E**) Aβ aggregation in SH-SY5Y cells was measured by thioflavin-T analysis. All experiments were performed in triplicate, and data are mean ± s.e.m. * *p* < 0.1, **** *p* < 0.0001.

The Aβ peptide, which plays a central role in AD progression, is neurotoxic. Aβ metabolism is regulated by at least two peptidases, IDE and NEP, which promote Aβ degradation within the brain [19,20,21]. We evaluated the levels of IDE and NEP expression in neuronal cells. Real-time PCR results revealed that ginsenoside F1 (1, 2.5, 5, and 10 μM) increased the levels of IDE and NEP mRNA in N2a (Figure 3A,B) and SH-SY5Y (Figure 4A,B) cells. Western blot analysis revealed that ginsenoside F1 (1, 2.5, 5, and 10 μM) caused an increase in the levels of IDE and NEP protein in N2a (Figure 3C,D) and SH-SY5Y (Figure 4C,D) cells. Ginsenoside F1 (2.5 μM) did not affect Aβ_1–42_ degradation in N2a (Figure 3E) or SH-SY5Y (Figure 4E) cells treated with IDE and NEP siRNA. Thus, ginsenoside F1 induces Aβ_1–42_ degradation by increasing the expression of Aβ-degrading peptidases.

### 3.4. Ginsenoside F1 Can Pass BBB

**Figure 5:**
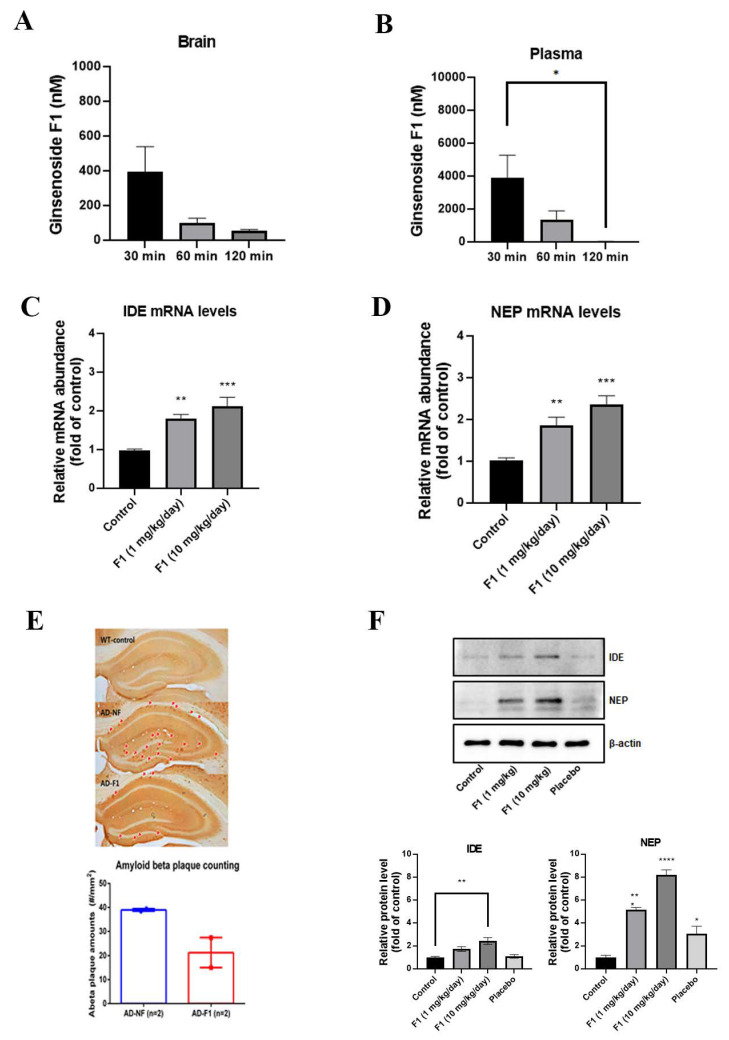
Ginsenoside F1 passes through the blood–brain barrier and reduces amyloid β (Aβ) plaque formation in vivo. Ginsenoside F1 (5 mg/kg) was administered to APP/PS1 mice by i.v. injection. (*n* = 5) Then, ginsenoside F1 was detected by LC-MS/MS analysis. (**A**) The concentrations of ginsenoside F1 in the brain were determined using LC-MS/MS. (**B**) The concentrations of ginsenoside F1 in the blood were determined using LC-MS/MS. All experiments were performed in triplicate, and the means and SDs are shown. Ginsenoside F1 (1, 10 mg/kg/day) was administered to mice by oral administration for four weeks. Then, Aβ plaques were detected using an immunohistochemistry stain. (**C**,**D**) mRNA transcriptional levels of IDE and NEP in the brain were measured using real-time PCR analysis. (**E**) Aβ plaques were detected using an immunohistochemistry stain. (**F**) Protein expression levels of IDE and NEP were detected using Western blotting. Data are mean ± s.e.m. * *p* < 0.1, ** *p* < 0.01, *** *p* < 0.001, **** *p* < 0.0001.

The use of effective, targeted drug-delivery systems for AD treatment is a challenge due to the presence of BBB. Ginsenoside Rg1 cannot pass through BBB, but whether ginsenoside F1 can pass is not known. Therefore, we investigated whether ginsenoside F1 could pass through BBB. We administered ginsenoside F1 (5 mg/kg) to mice by intravenous injection and measured the concentrations of ginsenoside F1 in the brain and plasma using LC-MS/MS. As shown in Figure 5A,B, 398, 104, and 43 nM of F1 were detected in the brain tissue; 3907, 1388, and 35 nM of F1 were detected in the plasma at 30 min, 1 h, and 2 h after i.v. injection, respectively.

### 3.5. Ginsenoside F1 Reduces the Aβ Plaque Formation through Aβ Peptidases in an AD Mouse Model

We detected Aβ plaques in the brains of APPswe/PSEN1dE9 double-transgenic mice (APP/PS1 mice) using immunohistochemical staining. Treatment with ginsenoside F1 reduced Aβ plaque formation in the hippocampal region of the treated group compared with the non-treated group (Figure 5E). In addition, studies have demonstrated that Aβ can be degraded by IDE and NEP [22,23,24]. Accordingly, our western blotting results confirmed that ginsenoside F1 increased both the mRNA levels of IDE and NEP (Figure 5C,D) and protein (Figure 5F) in the AD mouse model.

## 4. Discussion

AD pathogenesis is widely known to be driven by the production and deposition of the Aβ. Importantly, few studies have reported the preservative effect of ginseng on brain impairment via increasing the IDE and NEP system in mouse brain. To of our knowledge, this is the first research to provide evidence that ginsenoside F1 ameliorates AD symptoms through regulation IDE and NEP. To determine the underlying mechanism, we examined the protective effect of ginsenoside F1 on Aβ-induced cytotoxicity in neuronal cell lines and Aβ plaque formation in APP/PS1 mice. We have shown that IDE and NEP were upregulated at both the protein and mRNA levels in neuronal cell lines and APP/PS1 mice. Thus, we propose a novel mechanism by which ginsenoside F1 reduces Aβ level or Aβ plaques through the regulation of IDE and NEP in vitro and in vivo.

Many studies have demonstrated the role of IDE in the pathogenesis of AD, suggesting IDE may be a potential treatment target. IDE is observed in regions of the brain affected by AD and may be one of several proteases important for clearing Aβ plaques and extracellular Aβ peptides [25,26,27]. In addition, NEP has been implicated in the catabolism of Aβ peptides in AD. Lower levels of NEP mRNA and protein are related with increasing age and AD [28,29,30]. Also, NEP is able to degrade both monomeric and oligomeric forms of Aβ peptide. Hence, IDE and NEP levels may precisely impact the extracellular concentration of Aβ in brain tissue.

A few studies have suggested that Aβ oligomers diffuse readily through brain parenchyma and cause either selective synaptic dysfunction, neuronal loss, or both in the cortex and hippocampus, the two most affected regions of the brain in AD [4]. The accumulation of other Aβ peptides, which tend to form soluble oligomers, such as Aβ17–40/42 and Aβ25–35, has been noted in the brains of AD patients [31,32]. Increased levels of Aβ(1-42) are observed in the plasma and cerebral tissues of AD patients [33]. This connection provides that formation and metabolism of Aβ can affect another, and the end result will be inhibiting the progression of the AD. So, a mechanism to mediate the degrading of Aβ peptide by IDE and NEP may become a vital role in progression of the AD. Interestingly, ginsenoside F1 increased the expression levels of IDE and NEP protein and mRNA in neuronal cells and in an AD mouse model (Figure 3, Figure 4 and Figure 5). Our results suggest that ginsenoside F1 reduces Aβ plaques by inducing the expression of IDE and NEP.

Treating AD remains a challenge because the BBB prevents the entry of almost 98% of therapeutic agents, including drugs and genetic material, into the brain [34]. Despite recent advances toward the development of a therapy, no treatment can prevent, delay, or stop AD progression. APP/PS1 are double-transgenic mice expressing a chimeric mouse/human APP (Mo/HuAPP695swe) and a mutant human presenilin 1 (PS1-dE9), both directed at the neurons of the CNS. These two mutations are related with early onset AD and may be useful in studying neurological disorders of the brain, Aβ plaque formation or aging. Considerable efforts have been made to identify plant-derived, neuroprotective, antioxidants for treating neurodegenerative diseases [35,36]. Components of ginseng, including flavonoids, phenolic acids, and ginsenosides, are phytochemicals with a range of pharmacological effects [37,38,39,40]. These secondary metabolites are potent antioxidants and free radical scavengers; play anti-aging, neuroprotective, anti-inflammatory, neurotrophic, and anti-amyloidogenic roles in the CNS; and lower the decline in learning and memory [41,42]. Ginsenoside F1 was reported to improve memory in the APP/PS1 mouse model [43]. We detected ginsenoside F1 in brain and blood samples and confirm that it crosses the BBB. Apart from neurotoxicity, Aβ peptide may bind to certain receptors on glial cells and initiate immune response and neuroinflammation that contributes to the disease progress and severity. Importantly, our previous results indicated that F1 is able to attenuate glials-derived inflammation induced by oxidative stress [44]. Additionally, F1-enrich mixture effectively protect brain from scopolamine-induced cytotoxicity and memory impairments [45]. Thus, ginsenoside F1 is a promising candidate for targeting AD and other brain pathologies.

Amyloid fibrils in AD are formed from Aβ peptide, which arises in isoforms of different length. The residue peptide Aβ (1–40) and (1–42) represent the most abundant Aβ isoform in the brain and AD, respectively [46]. Aβ (1–42) peptide gives rise to the causal role in AD in the central nervous system via more neurotoxic aggregated form. Thus, targeting the inhibition of aggregation is one of most principal research objectives in the AD field. Herein, we reported that F1 could upregulate IDE and NEP and indirectly suppress the toxicity of Aβ (1–42) peptide. However, several further works are warranted to explore the potential binding capacity of F1 to Aβ peptide and to clearly explain how ginsenoside F1 regulates IDE and NEP. Additionally, AD formation model with late age would be another optimal challenge.

## 5. Conclusions

In conclusion, we observed that ginsenoside F1 exerts its beneficial effects by increasing IDE and NEP expression in an AD mouse model and propose its use to slow the progression of AD. Our study provides scientific evidence regarding the applicability of amyloid β treatment in patients with AD.

## Data Availability

Reported results can be found at the author.

## Supplementary Materials

The following are available online at https://www.mdpi.com/article/10.3390/life12010058/s1, Table S1: Oligonucleotide primers used for real-time PCR analysis. Table S2: Quantification of Western blot analysis.
