# Supplementary material for: Ginsenoside F1 Protects the Brain against Amyloid Beta-Induced Toxicity by Regulating IDE and NEP
Source: Life (Basel). 2022 Jan 1;12(1):58. doi: 10.3390/life12010058 (PMC8779788; doi:10.3390/life12010058)
Supplement: Supplementary file 1 [file life-12-00058-s001.zip › life-1465418-supplementary.pdf]

Table S1. Oligonucleotide primers used for real-time PCR analysis

Gene	Primer sequence (5′ – 3′)	
Human IDE	Forward primer :	TGCCCTAGACAGGTTTGCAC
	Reverse primer :	CTCCAGGCATCATTCATCACAT
Human NEP	Forward primer :	GATCAGCCTCTCGGTCCTTG
	Reverse primer :	TGTTTTGGATCAGTCGAGCAG
Human β-actin	Forward primer :	CTTCCTGGGCATGGAGTC
	Reverse primer :	AGCACTGTGTTGGCGTACAG
Mouse IDE	Forward primer :	CCGGCCATCCAGAGAATAGAA
	Reverse primer :	ACGGTATTCCCGTTTGTCTTCA
Mouse NEP	Forward primer :	GAGCCCCTTACTAGGCCTGTGT
	Reverse primer :	CTCGATTCAGACATAGGCTTTCTAAA
Mouse β-actin	Forward primer :	GGCTGTATTCCCCTCCATCG
	Reverse primer :	CCAGTTGGTAACAATGCCATGT

Table S2. Quantification of Western blot analysis

Cell lines	Ginsenoside F1 (μM)	Relative expression level (compare to β-actin)				
		0	1	2.5	5	10
Neuro 2 a	Extra IDE	0.060297262 ±0.00490975	0.056816089 ±0.00488863	0.072337993 ±0.00478263	0.070475163 ±0.00813391	0.068852188 ±0.00685621
	Intra IDE	0.064507161 ±0.00550610	0.099518976 ±0.00719678	0.083360618 ±0.00480450	0.102620078 ±0.01086344	0.053867472 ±0.00456915
	Extra NEP	0.140661029 ±0.16850375	0.03809949 ±0.00043241	0.052350845 ±0.00023394	0.050497053 ±0.00044273	0.041912808 ±0.00054702
	Intra NEP	0.031775973 ±0.00446764	0.017398984 ±0.00311185	0.058364351 ±0.00575529	0.09337957 ±0.01312376	0.081860579 ±0.00974111
SH-SY5Y	Extra IDE	0.016166548 ±0.00050224	0.014904626 ±0.00046054	0.017345037 ±0.00037325	0.042489446 ±0.00076560	0.069213963 ±0.00326720
	Intra IDE	0.097198707 ±0.11074645	0.184624739 ±0.20917092	0.136852251 ±0.15541457	0.18602652 ±0.21010480	0.169503936 ±0.19178243
	Extra NEP	0.040847406 ±0.0042865	0.045906719 ±0.00174226	0.061102445 ±0.0018077	0.103538657 ±0.0035553	0.065748978 ±0.00200912
	Intra NEP	0.058934134 ±0.0079543	0.072746579 ±0.00993095	0.068442722 ±0.008048348	0.069664999 ±0.00874212	0.043325967 ±0.006726617
